# Three Cases of Stent-Assisted Balloon-Induced Intimal Disruption and Relamination in Aortic Dissection Repair Effectively Treating Chronic Type B Aortic Dissection with False Lumen Aneurysm

**DOI:** 10.3400/avd.cr.25-00046

**Published:** 2025-09-20

**Authors:** Kanako Kobayashi, Naoki Fujimura, Ayaka Yu, Kyosuke Hosokawa, Yujiro Kawai, Takahito Itoh, Takahiro Shoji, Hirohisa Harada

**Affiliations:** 1 Department of Cardiovascular Surgery, Tokyo Saiseikai Central Hospital, Tokyo, Japan; 2 Department of Vascular Surgery, Tokyo Saiseikai Central Hospital, Tokyo, Japan; 3 Department of Emergency & Critical Care Medicine, Tokyo Saiseikai Central Hospital, Tokyo, Japan

**Keywords:** chronic type B aortic dissection, thoracic endovascular aortic repair, STABILISE

## Abstract

Successful thoracic endovascular aortic repair for chronic type B aortic dissection with an enlarged false lumen depends on complete exclusion of the false lumen. Stent-assisted balloon-induced intimal disruption and relamination in aortic dissection repair (STABILISE) creates a single lumen in the dissected thoracic and abdominal aorta by disrupting the intima. We report our experience in the treatment of 3 cases of chronic dissection using the STABILISE procedure at our hospital from December 2019 to May 2022. The STABILISE technique appears to be an effective procedure; however, further evaluation of risk factors for complications such as intraoperative aortic rupture is necessary.

## Introduction

Thoracoabdominal aortic replacement is the standard surgical treatment for chronic type B aortic dissection (TBAD) with an enlarged false lumen and aneurysm formation; however, this procedure remains challenging due to its high invasiveness and associated high mortality and morbidity rates, especially in high-risk patients, including the elderly.^1,2)^ Although thoracic endovascular aortic repair (TEVAR) is minimally invasive, mid- and long-term data have demonstrated higher reintervention rates in chronic TBAD treatment,^3)^ as residual retrograde flow from the false lumen into the aneurysm is associated with progressive aneurysmal enlargement. The success of the TEVAR for chronic TBAD depends on complete exclusion of the enlarged false lumen.^4)^

Stent-assisted balloon-induced intimal disruption and relamination in aortic dissection repair (STABILISE) is a procedure in which an aortic balloon catheter is dilated inside a stent graft and bare stent to disrupt the intima and eliminate the false lumen, resulting in formation of a single lumen within the dissected thoracoabdominal aorta.^5)^ The STABILISE technique was originally performed during the acute or subacute phase of TBAD.^6–8)^ At our institution, the STABILISE technique is part of a pre-planned strategy for treating chronic TBAD with aneurysmal degeneration. When persistent retrograde perfusion into the false lumen is anticipated and observed intraoperatively, the STABILISE technique is performed to achieve complete false lumen thrombosis and reduce the risk of reintervention. Herein, we present 3 cases of the STABILISE technique for chronic TBAD. Retrospective review of the STABILISE cases was approved by the Institutional Review Board at Saiseikai Central Hospital (2021-043).

## Case Report

### Case 1: 65-year-old man

The patient developed acute TBAD extending from the distal arch to the renal artery (RA) and was treated conservatively with outpatient follow-up. Nine years after the acute dissection, expansion of the dissected distal arch to 50 mm was observed. Because the patient had myasthenia gravis and had been receiving a low dose of steroids (prednisolone 5 mg/day), 1 debranch TEVAR was performed using TAG conformable thoracic stent grafts (TGU373715J and TGU312610J; W. L. Gore & Associates, Flagstaff, AZ, USA) and left common carotid artery (CCA) to left subclavian artery (SCA) bypass to close the entry tear. Two years after 1 debranch TEVAR (11 years following the initial dissection), the dissected distal arch expanded again (diameter, 61 mm), due to retrograde perfusion from the false lumen. The STABILISE technique was then chosen to control retrograde perfusion.

#### Preoperative computed tomography (CT)

**Fig. 1:**
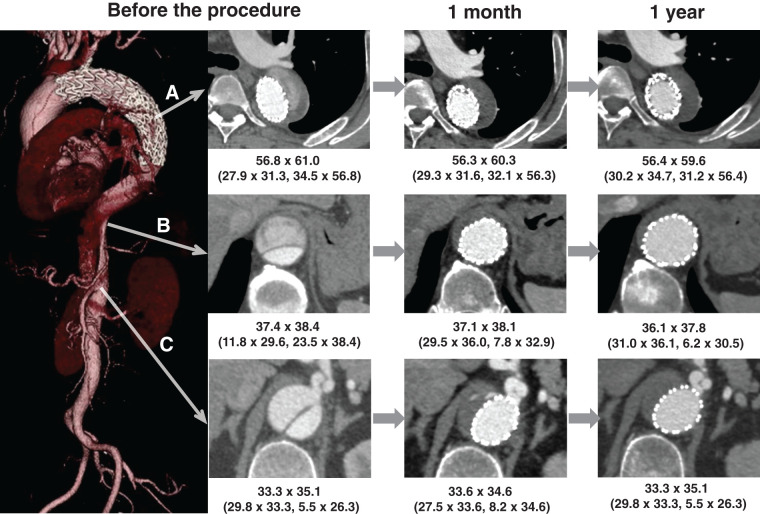
CT images of case 1 at 3 time points: preoperatively, 1 month, and 1 year after the STABILISE procedure. Aortic diameter (short axis × long axis, mm); TL and FL diameters are shown in parentheses. (**A**) Descending thoracic aorta at the level of the tracheal bifurcation. Preoperatively, FL expansion was observed due to retrograde flow; this resolved at 1 month. At 1 year, FL was thrombosed with aneurysm shrinkage. (**B**) Descending thoracic aorta at the level of thoracic vertebrae (T) 7 to 9. Preoperatively, a patent FL was present. At 1 month, TL was dilated and FL was obliterated. At 1 year, FL continued to be obliterated. (**C**) Abdominal aorta at the level of the celiac artery bifurcation. Preoperatively, the celiac artery originated from both TL and FL. At 1 month, FL thrombosed while the celiac artery perfusion from TL persisted. At 1 year, FL remained thrombosed. FL: false lumen; STABILISE: stent-assisted balloon-induced intimal disruption and relamination in aortic dissection repair; TL: true lumen; CT: computed tomography

CT showed a dissected aortic aneurysm (diameter, 61 mm) at the distal arch, with a false lumen extending to just below the RA. The celiac artery (CA) branched from both the true and false lumens, whereas the other abdominal branches originated from the true lumen (**Fig. 1**).

#### Operation

The left common femoral artery (CFA) was exposed via cutdown under general anesthesia. A Zenith Alpha thoracic endovascular graft (ZTA-P-38-217-W1; Cook Medical, Bloomington, IN, USA) was delivered and deployed with the distal end just above the CA. Next, a TXD bare metal stent (GZSD-46-164-2; Cook Medical) was delivered overlapping the aforementioned Zenith Alpha by 1 stent and deployed to cover the abdominal branches and end at the center of the abdominal aorta. As for the STABILISE technique, a Coda balloon catheter (Cook Medical) was delivered into the Zenith Alpha and inflated manually. After confirming the expansion of the Zenith Alpha Thoracic, the balloon was sequentially dilated multiple times from proximal to distal through the TXD bare stent. Post-procedure aortography confirmed dilatation of the true lumen and disappearance of the false lumen. Total operative time: 135 min.

#### Postoperative course

Postoperative CT imaging revealed no complications and disappearance of the false lumen (**Fig. 1**); therefore, the patient was discharged on postoperative day 7. CT images obtained 3 years after the surgery showed no enlargement of the distal arch aortic aneurysm, stable dilation of the true lumen, and almost complete disappearance of the false lumen from the descending thoracic aorta to the CA (**Fig. 1**).

### Case 2: 66-year-old man

The patient initially underwent partial arch (brachiocephalic artery and CCA) replacement for acute type A aortic dissection that extended from the ascending aorta to the left external iliac artery (EIA). At the time of the surgery, there was a 43-mm saccular aneurysm of the descending thoracic aorta. Because it had thrombosed after the initial surgery, the aneurysm was managed conservatively without intervention. However, CT imaging performed 2 years later revealed new perfusion inside the aneurysm from the false lumen with concomitant expansion. The patient had hemiplegia from a previous stroke, so we decided TEVAR was the best option.

#### Preoperative CT

**Fig. 2:**
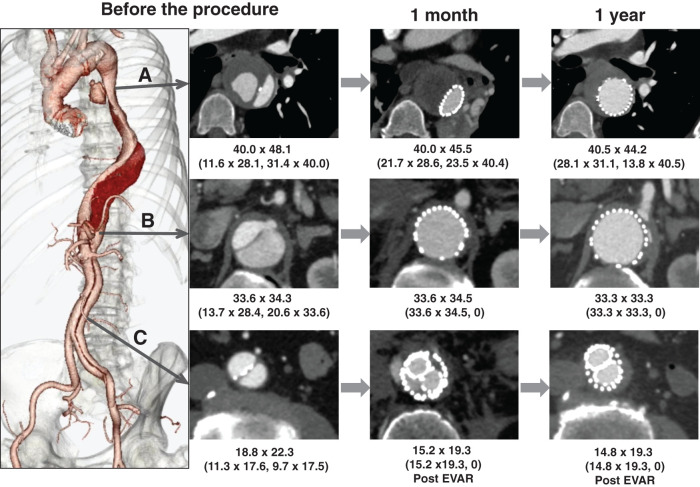
CT images of case 2 at 3 time points: preoperatively, 1 month, and 1 year after the STABILISE procedure. Aortic diameter (short axis × long axis, mm); TL and FL diameters are shown in parentheses. (**A**) Descending thoracic aorta at T4–T5 level. Preoperatively, aneurysm enlargement and retrograde flow from FL were observed in a previously thrombosed saccular aneurysm. At 1 month, FL was completely thrombosed. At 1 year, TL was dilated and FL had markedly decreased, with associated aneurysm shrinkage. (**B**) Abdominal aorta at the level of the celiac artery bifurcation. Preoperatively, FL was patent, and the celiac artery originated from compressed TL. At 1 month, TL was dilated and FL was obliterated; this was maintained at 1 year. (**C**) Abdominal aorta at the inferior mesenteric artery level. Preoperatively, FL was patent, and calcification was observed along the dissected intimal flap. Intraoperatively, aortic rupture occurred, and emergency EVAR was performed. At 1 month, FL had disappeared and the lumen outside EVAR limbs was thrombosed. At 1 year, the lumen outside EVAR had further decreased in size. FL: false lumen; TL: true lumen; STABILISE: stent-assisted balloon-induced intimal disruption and relamination in aortic dissection repair; CT: computed tomography; EVAR: endovascular aneurysm repair

CT revealed a new entry tear in the descending thoracic aorta with perfusion into a 48-mm saccular aneurysm of the descending thoracic aorta from the false lumen. The dissection extended to the left EIA, and all of the abdominal branches originated from the true lumen, and sporadic calcifications were observed in the dissected intima of the abdominal aorta (**Fig. 2**).

#### Operation

Under general anesthesia, the right CFA was exposed via cutdown. A Zenith TXD endovascular graft (ZTEG-2P-34-202-PF-D) was delivered and deployed from the origin of the left SCA to the descending thoracic aorta, followed by a Zenith Alpha distal extension (ESBE-40-81-T-PF-D) deployed below the Zenith TXD with 1.5 stent overlap. To prevent the bare stents from overlapping with each other around the abdominal branch, a TXD bare metal stent (GZSD-36-82-2) was deployed just above the aortic bifurcation, followed by another (GZSD-46-164-2), filling the space between the Zenith Alpha distal extension and the first TXD bare metal stent. Post-procedure aortography confirmed retrograde flow into the aneurysm from the false lumen, and the pre-planned STABILISE technique was performed to promote aortic remodeling and reduce the risk of reintervention. The TXD bare metal stent was manually dilated using a Coda balloon catheter from the distal end of the stent; however, abnormal behavior of the aortic margin was observed with an associated drop in blood pressure. Aortography revealed extravasation from the abdominal aorta (**Supplementary Video**). A Coda balloon catheter was promptly inserted and inflated, after which an Excluder AAA Endoprosthesis (RLT261412J and PLC141000J; W. L. Gore & Associates) was used for emergent endovascular aneurysm repair (EVAR). Post-EVAR angiography still showed extravasation due to false lumen flow. To control the flow in the false lumen, completion of the STABILISE technique was necessary and the Coda balloon catheter was manually dilatated inside the Zenith Alpha Distal Extension and sequentially downward from the descending aorta to the abdominal aorta. Retrograde angiography from the left CFA also revealed false lumen flow from the left EIA to the rupture site, which was treated by dilating the percutaneous transluminal angioplasty balloon catheter inside the Excluder limb in the left common iliac artery (CIA) to obliterate the false lumen, similar to the STABILISE technique. Post-procedure aortography confirmed no extravasation of the ruptured site, all abdominal branches visible without delay, and all false lumen flow obliterated from the descending aorta to the CIA, including flow to the aneurysm. Total operative time: 273 min.

#### Postoperative course

CT imaging on the first postoperative day revealed enlargement of the pseudoaneurysm at the site of rupture. Retrograde flow from the inferior mesenteric artery (IMA) was the bleeding source. The IMA was accessed via the superior mesenteric artery (SMA) and middle colic artery. Coil embolization and N-butyl cyanoacrylate injection were performed around the pseudoaneurysm and Excluder. The IMA origin was also embolized. After the procedure, the patient developed acute respiratory distress syndrome, and was placed on extracorporeal membrane oxygenation (ECMO). Fortunately, the patient recovered and was weaned off ECMO on the 6th postoperative day, extubated on the 7th, and discharged to his home on the 23rd. A CT 1 year after surgery confirmed the disappearance of the pseudoaneurysm at the rupture site, no further enlargement of the descending thoracic aortic aneurysm, dilation of the true lumen, and elimination of the false lumen from the descending thoracic aorta to the abdominal aorta (**Fig. 2**).

### Case 3: 50-year-old man

A patient with a history of acute TBAD 5 years prior was emergently transferred to our hospital with loss of consciousness due to repeated ventricular flutter. An implantable cardioverter-defibrillator was placed, and oral administration of amiodarone was continued; however, the patient’s cardiac function only improved to a moderate level. During the treatment, CT revealed a 58-mm dissecting aortic aneurysm of the distal arch, with the dissection extending from the arch to the right EIA. As the patient had chronic heart failure, TEVAR was selected as the best treatment.

#### Preoperative CT

**Fig. 3:**
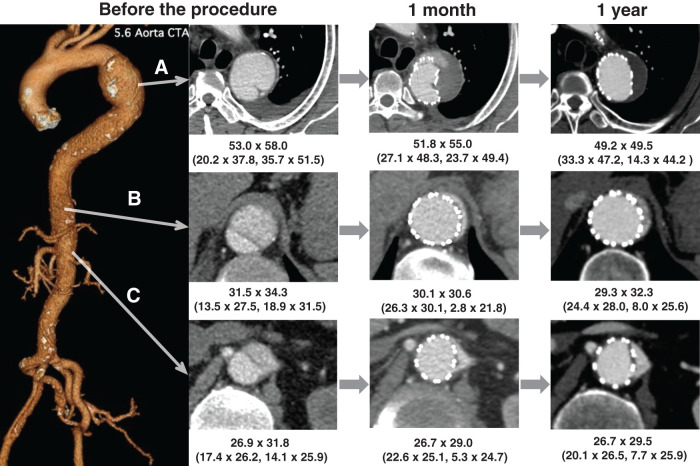
CT images of case 3 at 3 time points: preoperatively, 1 month, and 1 year after the STABILISE procedure. Aortic diameter (short axis × long axis, mm); TL and FL diameters are shown in parentheses. (**A**) Thoracic descending aortic aneurysm at T4–T5 level. Preoperatively, a chronic dissection with the primary entry tear located at the distal aortic arch and a 58-mm aneurysm was observed. At 1 month postoperatively, type 1A endoleak was still present. At 1 year, the endoleak had disappeared and aneurysm sac shrinkage was observed. (**B**) Descending thoracic aorta just above the celiac artery. Preoperatively, FL was patent and TL was compressed. At 1 month postoperatively, TL was dilated, but a small patent FL remained. At 1 year, FL is still patent, but decreased in size. (**C**) Abdominal aorta at the origin of the renal arteries. Preoperatively, the left renal artery branched from both TL and FL. At 1 month postoperatively, TL was dilated and perfusion to the left renal artery was preserved, although a small patent FL remained. At 1 year, FL was still present without evidence of enlargement, and TL had further expanded. FL: false lumen; STABILISE: stent-assisted balloon-induced intimal disruption and relamination in aortic dissection repair; TL: true lumen; CT: computed tomography

CT revealed a 58-mm dissecting aortic aneurysm at the distal arch. The primary entry point was located at the distal arch, and the false lumen extended to the right EIA. The SMA and right RA branched from the true lumen, the CA and IMA from the false lumen, and the left RA from both (**Fig. 3**).

#### Operation

The left CFA was percutaneously cannulated under general anesthesia using the Perclose ProGlide vascular closure system (Abbott Vascular, Santa Clara, CA, USA). A Zenith TXD endovascular graft (ZTEG-2P-34-202-PF-D) and Zenith Alpha distal extension (ESBE-36-77-T-PF-D) were deployed from the origin of the left SCA to the descending thoracic aorta. Next, 2 TXD bare metal stents (GZSD-36-164-2) were deployed so that they did not overlap around the abdominal branches. Post-placement aortography still showed retrograde perfusion of the aneurysm from the false lumen. In accordance with the preoperative plan, the STABILISE technique was then performed to promote aortic remodeling and reduce the risk of future reintervention. A Coda balloon catheter was delivered into the Zenith Alpha distal extension and manually inflated with caution. After confirming good dilation of the stent graft, the balloon was sequentially dilated distally to the end of the TXD bare metal stents. Although the area around the abdominal branch could not be completely dilated and a slight false lumen remained in the abdominal aorta, the procedure was terminated, as the false lumen of the descending thoracic aorta disappeared and the blood flow to the aneurysm in the distal arch was also obliterated (operative time, 132 min).

#### Postoperative course

Postoperative CT imaging revealed a small amount of type 1A endoleak; however, given no other complications, the patient was discharged on postoperative day 4. A CT 1 year after surgery revealed a residual false lumen; however, the type 1A endoleak and false lumen at the distal descending thoracic aorta disappeared, and no enlargement of the distal arch aortic aneurysm was confirmed (**Fig. 3**).

## Discussion

We have presented herein 3 cases in which the STABILISE technique was performed for the treatment of chronic TBAD. During the STABILISE technique, an aortic balloon catheter is dilated inside a stent graft and bare metal stent to forcefully destroy the dissected intima and eliminate the false lumen, thus converting the dissected aorta into a single lumen. The STABILISE technique is usually performed during the acute or subacute phase of TBAD,^6–8)^ with only a few reports of its use during the chronic stage.

In chronic stages of TBAD, the remodeling rate after primary entry closure alone has been reported to be limited, partly due to persistent retrograde flow into the false lumen. This may lead to gradual enlargement of the aneurysm over time and potentially necessitate reintervention. To address this concern and improve the likelihood of complete false lumen thrombosis, we employed the STABILISE technique. This approach has been reported to more reliably control false lumen perfusion than conventional TEVAR, with favorable outcomes in chronic cases.^9)^ In our 3 cases, complete obliteration of blood flow into the thoracic aneurysm was achieved. Given the technical challenges and unclear long-term outcomes associated with alternative methods such as the candy-plug, false lumen embolization, or treating all entry tears, the STABILISE technique may offer a promising option for managing chronic TBAD with a false lumen aneurysm.

However, 1 factor limiting the use of the STABILISE technique in the chronic stage of TBAD is that the descending aorta diameter is often very large in patients with chronic aortic dissection requiring treatment. When the diameter of the descending aorta exceeds 42 mm, the stent graft cannot seal the false lumen after the STABILISE technique is performed. In addition, the devices used were originally designed for acute and subacute dissections to seal entry tears and improve malperfusion. In the STABILISE technique for chronic TBAD, they are applied with a different therapeutic intent—to eliminate retrograde perfusion into the false lumen and promote thrombosis. This difference in intended use may represent an additional limitation in chronic TBAD. These problems may also affect the long-term result of the STABILISE technique as the aortic diameter may enlarge and cause reperfusion of the false lumen in the future, especially in the portion where TXD bare metal stents are placed. There have been no reports on long-term follow-ups of the STABILISE technique; therefore, further investigation is needed.

A more significant issue with the STABILISE technique in the chronic stage is that the dissected intima or flap is often fibrotic and hardened, making its disruption difficult. According to Faure et al.,^9)^ of the 17 patients who underwent the STABILISE technique for the treatment of chronic type B dissection, 2 had residual false lumens within 1 year after the acute dissection. Conversely, residual false lumens were not observed in patients treated more than 3 years after the initial dissection. This finding suggests that there is no direct relationship between the time since the onset of dissection and the degree of flap stiffness, and that it is hard to preoperatively evaluate the difficulty of achieving flap disruption.

The issue of flap hardening can cause overdilation of the aortic balloon catheter, and ultimately, vascular rupture, the most devastating complication of the STABILISE technique, as seen in case 2. The theory behind the STABILISE technique is that as the adventitia is the strongest and toughest structure of the arterial wall, the intima should be disrupted before the adventitia. In case 2, a sporadic calcification was observed at the flap, indicating over-stiffness. Furthermore, in the standard STABILISE technique, the disruption occurs first inside the stent graft to confirm safety, then using the flap tear caused by the first dilation, sequential dilation is performed distally. However, in case 2, the first dilation was performed at the distal end of the bare metal stent. These 2 factors probably affected the aortic rupture and should have been avoided. Subsequently, no cases of aortic ruptures have been reported at our hospital.

Considering all these issues and given the unclear long-term outcomes of the STABILISE technique, open surgical repair remains the first-line treatment for chronic TBAD at our hospital. STABILISE is reserved for high-risk cases where open surgical repair (OSR) is intolerable and complete false lumen exclusion is required, as in our 3 aneurysm cases, after multidisciplinary discussion. Theoretically, the STABILISE technique creates a distal landing zone, making the postoperative approach similar to that of TEVAR for a typical descending thoracic aortic aneurysm. Therefore, we intend to monitor the long-term progression of these cases.

## Conclusion

The STABILISE technique for the treatment of TBAD seems to be effective in obliterating the false lumen in the acute, subacute, and chronic stages. However, a complication of intraoperative aortic rupture was observed in our series, highlighting the need for further investigations to assess risk factors for complications.

## Supplementary Information

Supplementary Movie A Zenith TXD endovascular graft and a TXD bare metal stent were delivered and deployed from the origin of the left subclavian artery to just above the aortic bifurcation. Post-procedure aortography confirmed the presence of retrograde flow into the aneurysmal sac from the false lumen. As part of the STABILISE technique, the TXD bare metal stent was manually dilated using a Coda balloon catheter from the distal end of the stent; however, abnormal behavior was observed along the aortic margin with an associated drop in blood pressure. Aortography was immediately performed and revealed extravasation from the abdominal aorta.
